# Nutritional Requirements and Their Importance for Virulence of Pathogenic *Cryptococcus* Species

**DOI:** 10.3390/microorganisms5040065

**Published:** 2017-09-30

**Authors:** Rhys A. Watkins, Jason S. King, Simon A. Johnston

**Affiliations:** 1 Department of Infection, Immunity and Cardiovascular Disease, Medical School, University of Sheffield, Sheffield S10 2TN, UK; rawatkins1@sheffield.ac.uk; 2 The Bateson Centre, University of Sheffield, Sheffield S10 2TN, UK; jason.king@sheffield.ac.uk; 3 Department of Biomedical Sciences, University of Sheffield, Sheffield S10 2TN, UK

**Keywords:** *Cryptococcus neoformans*, *Cryptococcus gattii*, basidiomycete, fungal pathogens, evolution of virulence, host pathogen interactions, nutrition acquisition, nutritional restriction

## Abstract

*Cryptococcus* sp. are basidiomycete yeasts which can be found widely, free-living in the environment. Interactions with natural predators, such as amoebae in the soil, are thought to have promoted the development of adaptations enabling the organism to survive inside human macrophages. Infection with *Cryptococcus* in humans occurs following inhalation of desiccated yeast cells or spore particles and may result in fatal meningoencephalitis. Human disease is caused almost exclusively by the *Cryptococcus neoformans* species complex, which predominantly infects immunocompromised patients, and the *Cryptococcus gattii* species complex, which is capable of infecting immunocompetent individuals. The nutritional requirements of *Cryptococcus* are critical for its virulence in animals. *Cryptococcus* has evolved a broad range of nutrient acquisition strategies, many if not most of which also appear to contribute to its virulence, enabling infection of animal hosts. In this review, we summarise the current understanding of nutritional requirements and acquisition in *Cryptococcus* and offer perspectives to its evolution as a significant pathogen of humans.

## 1. Introduction

*Cryptococcus* is an environmental basidiomycete yeast that can cause meningitis and death in humans, mainly in the severely immunocompromised. The two major pathogenic groups of cryptococci are the *C. neoformans* species complex and *C. gattii* species complex. *C. gattii* are associated with primary infection in immunocompetent individuals but there is increasing evidence for *C. gattii* infection in the immunocompromised. In contrast, *C. neoformans* infection occurs in immunocompromised individuals e.g., HIV infection associated T cell depletion or immunosuppression following solid organ transplant [1,2,3]. 

Infective particles, such as spores and yeast cells are inhaled into the lungs [4,5,6]. In most immunocompetent hosts, these are thought to be either cleared by macrophages or contained in granulomas, leading to a period of latency or subclinical infection [7,8]. These focal lung lesions can be treated through antifungal drug therapy, or surgical excision [2,9]. However, if the infective organism is successful, an asymptomatic primary pulmonary infection can develop, which can be associated with the hilar lymph nodes [9]. 

Meningoencephalitis is the most common severe clinical manifestation of cryptococcal infection [10,11]. Early symptoms include headache, fever, confusion, or drowsiness. Seizures occasionally occur with *C. gattii* infection [2]. Symptoms of lung infection include fever, chest pain, and cough; chest X-ray often shows nodules or lesions in the lung. The presentation varies from asymptomatic infection to severe pneumonia with acute respiratory failure, and may signify reactivation of a latent infection [7] or progression of a primary infection [12].

The third most common site of cryptococcal infection is the skin. Although *Cryptococcus* can cause direct skin infection, the manifestation of disease in the skin usually indicates disseminated disease [13]. Other organs may also be affected in disseminated disease, such as the prostate gland, eyes or mouth [13]. Infection with *C. neoformans* is certainly not unique to humans and cases have been reported in a wide range of wild and domestic animals [14,15,16,17]. 

The transmission of cryptococcosis is rarely zoonotic [18,19,20] but may be iatrogenic [1,21]; most cases of this disease are most likely initiated by inhalation of airborne dry fungal cells or spores from an environmental source [4,5,6]. Isolates of *Cryptococcus neoformans* consisting of desiccated yeast cells or spores, measuring 0.6–3.5 μm in diameter, a size ideal for alveolar deposition after inhalation, have been described from aerosols generated from infected soil and pigeon droppings [4,22,23]. This supports the hypothesis that both desiccated yeast cells and basidiospores are pathogenic infectious forms of *C. neoformans* [6,14]. Air samples taken underneath flowering *Eucalyptus camaldulensis* trees have also captured cells of *C. gattii* [24,25]. During active growth, cryptococcal cells are too large to penetrate the human lung parenchyma. Whether derived from spores or from yeast cells, once inside a mammalian host all cryptococci transition to or maintain the yeast form [26] and are ingested by host phagocytes. Eventually, *Cryptococcus* may rupture phagocytes by lysis or it may escape via vomocytosis, leaving the host cell intact, before spreading to the central nervous system [27,28,29,30]. The ability of *C. neoformans* to exit cells non-lytically, without causing macrophage damage or death, avoids stimulation of the immune response and allows dissemination of the pathogen [28]. *C. neoformans* can also proliferate within immune cells and show remarkable adaptation during infection, including modulation of virulence mechanisms such as polysaccharide capsule expansion [31]. 

In this review, we summarise the current understanding of *Cryptococcus* nutritional requirements and acquisition in its life cycle and during infection. We first describe the biology of cryptococci, before discussing the varied nutritional requirements of cryptococci in the context of virulence and pathogenesis. Finally, we comment on the environmental niche of *Cryptococcus* and offer perspectives to its evolution as a significant pathogen of humans.

## 2. Description and Ecology

*Cryptococcus neoformans* is abundant in the environment with a saprophytic existence, yet uncommonly for a fungus, it can be highly pathogenic in animals and humans. *Cryptococcus neoformans* has a global distribution and exists as four antigenic serotypes A, B, C, and D [32]. Since its identification in 1894, the disease of cryptococcosis had been attributed to just one single fungal species, *Cryptococcus neoformans,* but in 1982 *C. neoformans* was subdivided into two varieties: *C. neoformans* var. neoformans (consisting of serotypes A and D) and *C. neoformans* var. gattii (serotypes B and C) [33]; further, using molecular techniques, *Cryptococcus neoformans* var. gattii was re-classified as a separate species, *Cryptococcus gattii* [34]. These two species, *Cryptococcus neoformans* and *Cryptococcus gattii* have different ecological and geographical distributions, with different virulence properties towards humans and are believed to have diverged from one another 37–40 million years ago [35]. Characterization of cryptococcal strains from around the world has since clarified the further subdivision of *C. neoformans* and *C. gattii* [36] although the suggested new nomenclature and classification may have some incongruities as, for example, many isolates from different lineages are capable of fusion with others during sexual reproduction [37]. It has therefore been suggested to refer to the “*Cryptococcus neoformans* species complex” and “*C. gattii* species complex” as an alternative, rather than creating more labels to define the species and the many strains therein [38]. In this review, when we refer to *C. neoformans* and *C. gattii* we are referring to the *C. neoformans* species complex and *C. gattii* species complex, respectively.

Study of the ecology of *C. gattii* and *C. neoformans* demonstrates that they can be found in distinct environments. *Cryptococcus gattii* is associated with the wood, bark, flowers, and leaves of Eucalyptus trees (*E. camaldulensis*), and is known to causes infection in Australian aborigines [39]. It is also found on other trees such as Douglas fir (*Pseudotsuga menziesii*) in California [40], carob (*Ceratonia silique*) and pine (*Pinus halepensis*) in the Mediterranean [41], or the mopane tree (*Colophospermum mopane*) in southern Africa [42]. *Cryptococcus neoformans* is found in soil and bird excreta [25]. The fungus may colonise the guano of birds in part due to the relatively low competition from bacteria [23]. *C. neoformans* and *C. gattii* both grow on pigeon guano but *C. gattii* is unable to mate efficiently in this environment, unlike *C. neoformans*, and suggest that pigeon guano is a realised ecological niche for *C. neoformans* but not for *C. gattii* [43]. Whilst birds may act as vectors for the dispersion of *Cryptococcus* they do not succumb to cryptococcosis as avian body temperature of 40–42 °C is too high to allow for disease progression [26,44,45]. Recent data has demonstrated that *C. neoformans* is in fact able to grow at bird body temperature but, unlike the case with mammalian phagocytes alone, when internalised by avian macrophages the yeast does not survive. [20]. Study of *C. neoformans* in Africa identified that most *C. neoformans* clinical infections were of the VNI genotype and were found in urban environments, where it would be expected that pigeon guano was common [46]. In contrast, rural sites were overwhelmingly positive for the VNB genotype and showed much greater genetic diversity. This finding is of significant interest as it suggests that the increased competition in the environment results in higher genetic diversity, while *C. neoformans* in the urban environment is not under the same selective pressure (see Section 5 below). However, our understanding of the natural environment of *Cryptococcus* is generally poor and there have been few in depth ecological sampling studies. A recent comparison of the location of *C. neoformans* and *C. gattii* in Columbia found *C. neoformans* commonly in arboreal sampling and found no sampling differences between *C. neoformans* and *C. gattii* [47]. Given the pivotal role of the ecology of environmental opportunistic pathogens in their virulence, this should be a focus of further research. 

## 3. Life Cycle

*Cryptococcus neoformans* can reproduce asexually by budding, by monokaryotic fruiting, or by mating sexually. Although *C. neoformans* can survive in the mammalian host, where it causes infection, it is naturally found in soil and arboreal environments [24,25,48]. The fungus has several morphological forms in nature but *C. neoformans* is usually isolated from patients, from laboratory cultures and from the environment as a budding yeast [40]. 

### 3.1. Asexual Reproduction

Mitotic reproduction by budding during yeast growth results in two independent cells per mitosis and provides the fastest mode of population increase [49]. The regulation of cell cycle is well conserved with the evolutionarily distant yeast *Saccharomyces cerevisiae*. Exposure to the antifungal drug fluconazole can cause defects in cytokinesis, resulting in multimeric cells that are better able to proliferate in the presence of fluconazole [50]. 

### 3.2. Sexual Reproduction and Monokaryotic Fruiting

*Cryptococcus* exhibits multi-cellular filamentous growth form as a result of sexual reproduction or by monokaryotic fruiting. Filamentation, where contiguous cells are partitioned by septa to form hyphae or pseudo-hyphae, confers wider nutrient scavenging and the ability to produce spores. Hyphal forms are not found in human infections [51] perhaps as the production of hyphae is strongly antigenic, stimulating clearance by the host [52]. 

The sexual cycle for *C. neoformans* involves fusion of haploid cells of opposite mating type (MAT**a** and MATα) to produce dikaryotic filaments [53]. When cells of opposite mating type are in close proximity, MATα cells respond to a MAT**a** peptide pheromone by developing conjugation tubes, while MAT**a** cells enlarge [54]. The conjugation tubes fuse to the swollen MAT**a** cells, forming heterokaryotic hyphae that subsequently develop basidia at their tips. MATα and MAT**a** nuclei fuse in the basidia, and undergo meiosis to produce haploid spores. Clinical and environmental isolates are usually haploid, possessing one of two mating type alleles, MAT**a** or MATα, although most clinical isolates are of the MATα mating type. Congenic MAT**a** and MATα strains are equally virulent [53] yet in co-infections with both mating types, the α strain is the more likely to enter the central nervous system [55], potentially explaining its clinical prevalence. Besides the stimulus of mating pheromone, several other factors are known to encourage hyphal growth, including ambient temperatures, nitrogen starvation, dehydrated substrates, darkness, and the presence of metals such as copper [56,57]. High concentration of CO_2_ and temperatures above 37 °C, characteristic of human host conditions, inhibit filamentous growth and may therefore suppress the transition from yeast to hyphal forms in infection [58]. Sexual reproduction in this fungus has been shown to generate short-term variation by increasing the incidence of aneuploidy, which can result in rapid adaptive phenotypic and genotypic evolution, such as the development of antifungal drug resistance [59]. 

*C. neoformans* strains can also reproduce through monokaryotic fruiting. In response to nutrient limitation, α cells in particular, can generate filaments by asexual fruiting and produce spores. The hyphal cells from monokaryotic fruiting differ from those produced through sexual reproduction by being mononucleate and diploid with unfused clamp connections; in sexual reproduction the hyphal cells are binucleate and haploid, joined by fused clamp connections [40]. 

### 3.3. Phenotypic Switching and Titan Cells

Phenotypic switching is reversible and occurs in adaptation to a new environment such as to escape recognition by the host immune system [60]. For example, *Candida albicans* is a dimorphic fungus, and the morphological switch between the yeast phase and the hyphal phase is considered to be its main virulence factor [61]. During infection, *C. neoformans* is almost always observed in the yeast form and is only occasionally found as the filamentous form. The host environment induces metabolic alterations in *C. neoformans*, as well as changes in the size and structure of the capsule [62,63,64] that may help also in evading the host immune response. Phenotypic switching in *Cryptococcus* has been demonstrated in vivo following serial passage through mice, revealing an increase in virulence caused by changes to the structure of the cell wall and/or the capsule [65,66]. 

A dramatic adaptation to the host is the formation of vastly enlarged yeast cells by *Cryptococcus*, first described in human infection and subsequently studied in the lungs of infected mice [64,67]. Titan cells have several characteristics that differentiate them from normal-size cells and promote disease by preventing clearance from the lung. Titan cells can be as large as 50 μm to 100 μm [68] and are thus too large to be phagocytosed by lung phagocytes and inhibit the phagocytosis of normal sized cryptococcal cells [68,69], with highly cross-linked capsules [64]. Titan cell walls are 30 to 50 times thicker than the normal *Cryptococcus* cell wall and are highly resistant to oxidative and nitrosative stress [64,67]. The polyploidy observed in titan cells is also reported to enhance their genetic adaptation to the stressful host environment, resulting in increased within-host survival [70]. In addition to the large titan cells, unusually small cryptococcal cells have also been observed [71,72]. These so-called micro cells are only 2–4 μm in size, with a thickened cell wall. They may be adapted for growth in macrophages but there is little information available about this form. 

The mechanisms of titan cell induction are still poorly understood but they form in response to activation of mating pheromone receptor Ste3**a,** and host temperature seems to be important in the generation of titan cells [67,73]. Due to their immense size, titan cells appear to be unable to pass to the brain [67,68]. However, the generation of titan cells promotes the spread of the normal-sized cryptococcal cells from the lungs to other tissues [68] and it has been estimated that the dissemination of *C. neoformans* to the CNS is increased by up to three hundred-fold by the production of titan cells [68]. In addition, mouse strain alters titan cell formation and correlates with a higher titre in IgE, although the mechanism of this effect is unknown [74]. Therefore, while titan cells use multiple mechanisms to avoid capture and killing by immune cells, how titan cell formation has evolved is an intriguing and unanswered question. While several nutritional factors have been implicated in titan cell formation (described in the corresponding sections below), how widespread titan cell formation is among yeasts and how the environmental niche of *Cryptococcus* contributes to regulation is not known. 

## 4. Nutrition in the Environment and Virulence in the Host

In this section, we relate nutrition with virulence separately for carbon, amino acid, melanin, lipid, metal ion, and phosphate metabolism. We will not dwell on the signal transduction systems in *C. neoformans* as they are generally well-conserved with those of other fungi, but the *C. neoformans* genome has revealed missing components in several signalling cascades, identified in non-pathogenic fungi. This is likely due to sequence divergence and many alternative signalling proteins have been identified but are yet to be placed within specific signalling cascades [49]. Growth at mammalian body temperature is independently controlled by the Ras (Ras1/2) and Ca^2+^-calcineurin pathways and the Mpk1 MAPK pathway is believed to regulate cell-wall integrity and growth at high temperature (37 °C) and therefore virulence [75]. The cyclic AMP (cAMP)/protein kinase A (PKA) pathway controls mating, as well as capsule and melanin production [56,76,77,78].

### 4.1. Carbon Utilisation and Metabolism

The carbon sources of *C. neoformans* and *C. gattii* in the environment are not known but due to its apparent saprophytic existence like other *Cryptococcus* sp. it is likely due to the breakdown of complex polysaccharides from decaying matter. *C. neoformans* appears unable to grow on cellulose or lignin as a carbon source despite its association with decaying plant matter and may therefore rely on the action of other microbes to release usable carbon sources [79]. *C. neoformans* grows well on fructose, glucose, and mannan monosaccharides but with an unusual requirement for peroxisome function [80]. Creatinine is not used as a source of carbon by *C. neoformans*, but is used as a source of nitrogen [81]. *C. neoformans* can use xylose, mannose, and mannitol as a sole carbon source [82]. Cherniak et al. used nuclear magnetic resonance spectroscopy (NMR) to study the assimilation of radiolabelled [1-13C]Xyl, [1-13C]Man, and [1-13C]mannitol mannose and their incorporation into capsular glucuronoxylomannan (GXM) [82]. The carbon chains of both mannose and mannitol are incorporated into capsular polysaccharide whilst the carbon chain of xylose was not incorporated intact by the yeast and it was suggested that xylose is assimilated through the pentophosphate pathway (hexose monophosphate shunt). Deletion of the two *C. neoformans* mannan transporters is not lethal but results in avirulence [83]. How *C. neoformans* is tolerant of the lack of specific mannan transporters is not known but it is possible that other sugar transporters may be able to transport sufficient mannose for survival. Metabolism of the C2 carboxylic acid glyoxylate by isocitrate lyase and malate synthase are required for growth on acetate but are dispensable for growth in animal infections [79]. Isolation of conditional mutants in essential genes of *C. neoformans* identified an enzyme (isopentenyl diphosphate:dimethylallyl diphosphate isomerase) in the isoprenoid pathway [84]. Isoprenoids are associated with stress responses and the mutant showed reduced stress tolerances. 

The endogenous carbohydrates of fungi comprise several monosaccharides such as glucose, acyclic sugar alcohols, or polyols and disaccharides, including trehalose [85]. Trehalose is produced by bacteria, fungi, protists, plants and invertebrates but has not been isolated from mammals. Trehalose may be used as an energy source but has primarily been described as a thermotolerance factor, protecting against protein denaturation. However, in *C. neoformans* mutations in the trehalose synthesis pathways have been shown to be essential for virulence in animals and while thermotolerance is a critical factor in *C. neoformans* and *C. gattii* pathogenesis, trehelose synthesis is likely to be of broader importance for tolerating the stress of animal infection [86]. Fungi also produce an assortment of storage, structural, and interactive polysaccharides, such as glycogen and various glucans; the glycol-proteins chitin, chitosan, mannans, and lectins [85]. Chitin is major component of the cell wall while mannan is required for, in addition to the cell wall, polysaccharide capsule, glycan lipids, and GPI anchor production [83]. Polyols such as glycerol and mannitol are widespread throughout the fungal kingdom and are multifunctional, acting as energy stores, as translocatory substances, or as intracellular reducing and osmoregulatory agents [87,88]. *C. neoformans* produces large amounts of mannitol in culture and in infected animals [89] yet its significance is uncertain. Wong et al. suggested that mannitol may protect the pathogen from free radicals and oxidative destruction by phagocytes [88]. It is also possible that mannitol could increase the tonicity of heavily infected tissues and, in the brain, may contribute to the development of cerebral oedema. An irradiated *C. neoformans* strain, producing low levels of mannitol (Cn MLP) was more susceptible to growth inhibition and killing by heat and high NaCl concentrations than a wild type *C. neoformans* strain [89]. Virulence in mice was also seen to be reduced in low mannitol producing strains and it was hypothesised that high intracellular mannitol levels may protect *C. neoformans* from the deleterious effects of heat and osmotic stresses, in the same way as high intracellular glycerol or D-arabinitol levels protect other fungi from similar stresses [87,89]. 

Arguably the most important and defining aspect of carbon metabolism in pathogenic cryptococci is the production and regulation of its polysaccharide capsule. The polysaccharide capsule surrounds the cell body of *Cryptococcus neoformans* and is comprised of 90–95% glucuronoxylomannan (GXM) and 5–8% galactoxylomannan (GalXM), together with a small amount (< 1%) of mannoproteins (MP) [63,82,90,91,92,93,94]. *C. neoformans* acapsular mutants are avirulent in mice [95]. In the external environment, the polysaccharide capsule of *Cryptococcus* might serve to prevent dehydration of the cells [96] but the capsule is recognised as one of the key virulence factors of pathogenic cryptococci. GXM is synthesised intracellularly and is secreted via exocytosis [97]. Capsule growth closely follows the cell cycle [98,99]. *Cryptococcus neoformans* can modify the size and structure of its capsule in response to various stimuli. Iron concentration, CO_2_, and serum are important signals affecting capsule growth. Iron limitation results in capsule growth [100] and a CO_2_-enriched atmosphere was shown to significantly increase the capsule size [101]. Both iron limitation and CO_2_ availability influence capsule size and these conditions may be encountered by the fungus during lung colonization. Serum or serum with CO_2_ encourages capsule enlargement [102], a phenomenon also observed during animal infection. Conversely, a dramatic decrease in capsule size is stimulated by high osmotic pressure, high salt (NaCl) concentration or the binding of a large quantity of antibody to the capsule [103,104]. Capsule enlargement can be observed after a few hours of infection in mice [72] and is also known to be stimulated in the presence of mannitol [105]. The structure of the capsule is very variable, depending on the strain and environmental conditions and it can also change during the course of an infection [106]. That various capsule phenotypes are observed in different organs indicates that this plasticity in *C. neoformans* to alter its capsule is biologically important; progress is being made in deciphering the signal transduction networks regulating the *C. neoformans* capsule [107]. Many of the capsule inducing factors found in in vitro and in vivo infection are likely to occur in the environment (e.g., salt stress/osmotic pressure/iron limitation) but cryptococci isolated from the environment typically have very small capsules. However, in vitro recapitulation of environmental biotic stress with incubation of amoebae and *Cryptococcus neoformans* results in increased capsule size [108]. 

### 4.2. Melanin

Melanin is a brown-black pigment and *C. neoformans* cells are often found melanised in bird guano [43,109]. *C. neoformans* and *C. gattii* can produce melanin from catecholamine precursors amino phenols, diaminobenzenes, and indoles, using diphenol oxidases with a wide substrate specificity [110]. As a free-radical scavenger, melanin protects *C. neoformans* against oxidants in vitro [111] and it has been shown that *C. neoformans* cells which produce melanin are more resistant to nitrogen- and oxygen-derived oxidants than non-melanised cells [111]. Melanin protects *Cryptococcus* from toxic free radicals that are produced by the host defence system [112,113]; it has also been shown to help defend the fungus against micro-biocidal proteins and antibiotics [114,115] and to alter the cellular charge of fungal cells, rendering them more resistant to phagocytosis [116]. Melanin is strongly associated with virulence and mutants of *C. neoformans* lacking melanin are less virulent in mice when compared to wild-type strains [117]. Other studies, inoculating mice with high and low melanin content strains of *Cryptococcus*, demonstrated that melanin inhibited host recognition of the pathogen, down-regulating the T cell-mediated immunity [118], confirming that melanin is essential for virulence. Interestingly, melanised forms of fungi, including *C. neoformans*, are also highly radiation resistant. Irradiated melanised *C. neoformans* showed increased metabolic activity, faster growth, and increased dry weight biomass than irradiated non-melanised cells [119,120]. In addition, melanin is involved in the reduction of iron prior to its uptake [121] and the synthesis of melanin is also dependent on iron levels [122]. 

### 4.3. Amino Acid Assimilation

Amino acids can be acquired by uptake, biosynthesis, or recycling. Although the mechanisms of amino acid acquisition by membrane bound permeases and recycling remains poorly understood in *C. neoformans*, this has been extensively explored in *S. cerevisiae* [123]. Recently, investigation of the tryptophan biosynthetic pathways in *C. neoformans* found eight potential global permeases and two methionine permease homologues. In other fungi, these global permeases are known to respond to nutritional status such as the quality of nitrogen and carbon sources, amino acid availability in the environment, and nutritional deprivation (Global Amino Acid Control) [124]. Interestingly, in *C. neoformans* only 8 amino acid permeases have been identified, far fewer than *S. cerevisiae* that has 24 permeases [123]. Contrary to the case of *S. cerevisiae, C. neoformans* thus appears more dependent upon biosynthesis than uptake [124] and recent reannotation of the *C. gattii* genome has demonstrated also that there is strong expression of amino acid synthetic genes during lung infection in mice [125]. Whilst the methionine pathway is not essential for growth it has a strong impact on cryptococcal virulence suggesting it is required under nutrient restricted conditions or under stress [126]. The threonine and tryptophan biosynthetic pathway are essential in *C. neoformans* [127].

Chang et al. identified three d-amino acid oxidase (DAO) genes in both *C. neoformans* H99 and *C. gattii* R265 strains and showed that each gene has a different role in d-amino acid utilization [128]. In *C. gattii* R265, the DAO2 gene was reported to have a role on d-amino acid assimilation, as deletion of this gene resulted in retarded growth on eleven d-amino acids. Although H99 grows only poorly on d-amino acids, deletion of the DAO1 or DAO3 genes exacerbated this effect, whilst overexpression of DAO2 or DAO3 enabled robust growth on several d-amino acids, implying that the DAO genes in H99 are normally insufficiently expressed for growth on d-amino acids. Creatinine can be used as the sole source of nitrogen by both *C. neoformans* and *C. gattii*, but the enzyme, creatinine deiminase, responsible for creatinine utilization, is regulated differently in the two species [80]. Creatinine deiminase is repressed by ammonia in *C. neoformans* but this is not the case in *C. gattii*. *C. neoformans* can use glycine only as a source of nitrogen whilst *C. gattii* can use glycine as both a carbon and a nitrogen source [129]. l-canavanine, that competes for l-arginine incorporation, is tolerated and assimilated by *C. gattii* while it is inhibitory to *C. neoformans* and therefore, together with glycine utilisation, forms the basis for selective media [130] *C. neoformans* can assimilate uric acid as a nitrogen source, which requires urease, a nitrogen-scavenging enzyme which is important in central nervous system invasion [131,132]. Screening of the *C. neoformans* genome revealed the existence of only one positively acting nitrogen regulatory GATA factor: Gat1 [133]. Gat1 mediates nitrogen metabolite repression and is required for utilization of ammonium and most nitrogen sources. This gene is also involved in capsule synthesis and the regulation of mating, melanin production, and growth [133]. 

### 4.4. Metal Homeostasis in Cryptococci

The regulation of essential metals such as iron, zinc, and copper is crucial to microbes during pathogenic interactions with a host [134]. Iron is vital as a cofactor of various enzymes, oxygen carriers, and electron-transfer systems involved in many fundamental cellular functions [135]. The effects of iron limitation on *C. neoformans* have been known for many years, as one important consequence is the enhancement of capsule formation [100]. Despite their different fungal groupings, *Cryptococcus neoformans* and *S. cerevisiae* share many similarities in metal homeostasis. Both utilise Fe(III) reductases as their major means of acquiring iron [136,137]. Although they do not synthesize siderophores, the two yeasts can extract iron from siderophores formed by other microorganisms [134,137]. At the same time, *C. neoformans* and *S. cerevisiae* can both use non-enzymatic means of reducing iron [100,121,137]. The non-enzymatic reduction of Fe(III) was observed with secreted 3-hydroxyanthranilic acid and melanin [121]. *C. neoformans* produces melanin by the oxidation of catecholamines, representing a natural means of iron recruitment [121].

Copper serves as a catalytic and structural cofactor for enzymes involved in energy generation, and processes such as iron acquisition, oxygen transport, and cellular metabolism [134]. It is also known to be involved in melanin synthesis and polysaccharide capsule production in *C. neoformans* [134,138]. Zinc homeostasis is essential for fungal growth as this metal is structurally critical for numerous enzymes and is a co-factor in the functionality of a wide variety of proteins [139]. It has been shown in *C. gattii* that disruption of the ZAP1 gene, which encodes a zinc sensor and transcription factor, reduces the virulence of the pathogen [140]. *Cryptococcus* uses carbonic anhydrases, which are zinc proteins, to convert CO_2_ to HCO_3_. Nickel is a cofactor for urease. As with urease-deficient strains, cryptococci with deficient nickel homeostasis show impaired dissemination following pulmonary infection [131,141]. 

### 4.5. Lipid Metabolism in Growth and Virulence

Lipid metabolism is essential for varied signalling pathways as well as for energy production and the maintenance of the plasma and intracellular membranes. Furthermore, production of the fungal lipid ergosterol (that serves the role of cholesterol in mammalian cells) is the target of azoles and amphotericin B antifungal agents, and several fungal lipids are distinct from those found in mammals. Fatty acid metabolism appears essential to *C. neoformans* as attempts to delete either of the identified fatty acid synthase genes were unsuccessful [142]. Cryptococci possess acetate kinases that are used by fungi to utilise short chain fatty acids as a carbon source [143] however fatty acid transporters in *C. neoformans* have yet to be identified. *C. neoformans* has a high triacylglycerol content, which increases in stationary phase growth and may serve as an energy store when under nutritional stress [144]. Diacylglycerol (DAG) is an important second messenger that activates protein kinase C. This pathway is conserved in *C. neoformans* and is critical for regulation of cell wall composition [145]. Ceramide is another lipid second messenger found in *Cryptococcus* that is metabolised to DAG by inositol-phosphorylceramide synthase [146]. Inositol-phosphorylceramide synthase is required for virulence and deletion of the enzyme leads to poor growth in macrophages and low pH [147]. Differences in sphingolipids and ergosterol levels between *C. neoformans*, *C. gattii*, and the rarely pathogenic *Cryptococcus albidus* and *Cryptococcus laurentii* have been observed but the details of their importance in cryptococcal biology is yet to be ascertained [148]. Lipid modification of proteins has been studied in *C. neoformans*, particularly modifications of the small GTPases, but not at all in *C. gattii* [149,150,151,152]. Mutation of S-acyl transferase results in dramatic morphological changes that are highly detrimental to stress tolerance and virulence [150,151]. Farnesyltransferase β-subunit is not required for budding growth except at high temperature but is required for sexual reproduction and virulence [152,153]. Studies of *C. neoformans* indicate, as for several other parasitic microorganisms, that phospholipases contribute to host cell penetration, injury, and lysis [154,155]. Mice inoculated with four strains of *C. neoformans* expressing high, intermediate, or low phospholipase activity and the subsequent infection levels quantified revealing a positive correlation between phospholipase activity, capsule size, and virulence [156]. Both *C. neoformans* and *C. gattii* have enzymes with phospholipase B, lysophospholipase, and lysophospholipase transacylase activity [157]. The best characterised enzyme is PLB1 which is required for virulence and growth in macrophages [158,159]. Phospholipids in the environment may induce titan cell formation and PLB1 has also been implicated in cell size regulation in *C. neoformans* [64,159]. 

Many fundamental aspects of lipid metabolism in *Cryptococcus* are still unknown. In particular, metabolic flux of lipid synthetic pathways and how these are modulated during infection are poorly characterised [148]. Lipid metabolomic studies of cryptococcal mutants and strains would be highly beneficial in our understanding of pathogenesis and the fundamental biology of cryptococci. 

### 4.6. Phosphate Uptake

Phosphate is essential for many cellular functions from kinase signalling to energy production and storage. *Cryptococcus*, like other pathogenic fungi examined, has a conserved phosphate uptake pathway that is absent in human cells [160]. Deletion of the transcriptional regulator of acquisition, Pho4, results in a failure to grow in the absence of added phosphate and with phosphate in alkaline and host (pH 7.4) conditions [161]. Thus, in murine infection the *PHO4* mutant fails to thrive and demonstrates reduced lung growth and less dissemination to the brain. Furthermore, cells defective in phosphate uptake have reduced capsule and melanin production and exhibit larger, irregular shaped cells [162]. How other cryptococcal species differ in phosphate uptake and how these mechanisms relate to their environmental niche is unknown.

## 5. Nutrition Acquisition in the Wider *Cryptococcus* Genus and Other Basidiomycota

Basidiomycete fungi are widespread in the environment and interspecies competition for nutrients is a factor in their survival. As part of the saprophytic biome, nutrient acquisition by basidiomycetes and the wider Cryptococcus genus has been the subject of significant research. Cellobiosan is an anhydrosugar, produced from the burning of biomass and which is then available to soil-dwelling microbes and Cryptococcal species are capable of utilising cellobiosan as a sole carbon source [163]. Previously, Middelhoven et al. had shown that several yeasts taken from soil could use uric acid, monoamines, and diamines as a sole carbon source [164]. The fruit pathogen *Cryptococcus laurentii* can use ethanolamine whilst other basidiomycete yeasts such as *Trichosporon aquatile* or *Trichosporon brassicae*, as well as *C. laurentii*, could also assimilate the polyamines spermine and spermidine. *Cryptococcus cerealis* is found in pig feed fermentations, indicating that it can tolerate low oxygen levels for respiration and may even have a role in reducing the oxygen content of feeds in the fermentation process [165]. Cryptococcal strains occurring on plants demonstrate proteolytic, lipolytic, and beta-glucosidase activity, with the broadest activities seen in yeasts associated with leaves [166]. This corresponds with earlier observations that basidiomycete yeasts are more proteolytic in nature than ascomycetes [167]. Although there is normally inter- and intra-species competition between organisms, the mycorrhizal fungus *Glomus mosseae* and several soil yeasts such as *Cryptococcus laurentii*, *C. aerius*, or *Candida sake* often interact synergistically and may improve the growth of plants by increasing the availability of nitrogen and phosphorous to the plant root system [168,169]. Under nitrogen-excess conditions the yeast *Cryptococcus curvatus* increases biomass and reduces lipid production. However, under nitrogen-limited conditions, *C. curvatus* can accumulate high levels of intra-cellular total sugars (ITS) if grown on lactose or sucrose as substrate. As nitrogen from the medium becomes exhausted, and ITS and lactose are consumed, lipids, mainly palmitic, stearic, and linoleic acids accumulate [170]. These single cell oils (SCOs), are produced in greater quantity on lactose as opposed to sucrose media and have great biotechnological significance.

## 6. Evolution of Cryptococcal Virulence in the Environment

Virulence of *Cryptococcus* to the host has been defined as a microbial characteristic that is expressed only in a susceptible host [37]. Of the 322 species listed within the *Cryptococcus* genus only seven are recognised as being pathogenic [171]. *Cryptococcus* life cycle is not dependent on infection of an animal host yet *C. neoformans* has a surprising capacity to infect and cause disease in a range of animal hosts, including humans, other mammals, birds, and reptiles. *Cryptococcus neoformans* has co-evolved over millions of years with free-living environmental phagocyte predators, such as amoebae, protozoa, nematodes, mites, and insects [172,173,174,175,176,177] and this association has led to the hypothesis that virulence of this fungus may have evolved and be maintained via selection imposed by exposure to such environmental predators [174,178]. Given the known natural history of *Cryptococcus* places them firmly in a saprophytic niche we can assume that they will have evolved extensive strategies to avoid predatory phagocytes. As mammalian phagocytes may originate from common environmental ancestors, it is possible that the roles of higher animal host phagocytes against *C. neoformans* reflect the complex interactions between the fungus and phagocyte predators in the environment [175]. *Acanthamoeba castellanii* is a common amoeba in soil and has been extensively studied as a model phagocyte and as a model of metazoan cell biology. Studies of *C. neoformans* interactions with *Acanthamoeba* demonstrated that following phagocytosis Cryptococci were able to replicate, killing the amoeba. Intriguingly, acapsular strains did not survive after phagocytosis and support the supposition that capsule may have evolved to avoid predatory phagocytes [178]. Melanization also appears to enhance fungal survival and a *C. neoformans* phospholipase mutant had a decreased replication rate in amoebae compared with wild-type phospholipase-producing strains as seen in mammalian macrophages [159]. Such characteristics are recognised as contributing to mammalian virulence. Many *C. neoformans* genes that are required for disease in humans are also required for infection, survival, and killing of amoebae, nematodes, and insects [178,179,180,181]. In addition, passage of an encapsulated *C. neoformans* strain through the social amoeba *Dictyostelium discoideum* was shown to increase yeast virulence in mice, with enlargement of capsules and faster time to melanisation [108], as has also been demonstrated in murine models [65,66,182]. This highlights that the possession of a polysaccharide capsule, production of melanin and phospholipase are advantageous to *C. neoformans* as virulence factors in a variety of animal and environmental hosts [174] and strongly suggests that predation by environmental organisms may have consequentially led to the evolution of and maintenance of *C. neoformans* virulence [173].

## 7. Summary and Perspectives

In addition to furthering our understanding of virulence of pathogenic fungi, understanding nutritional requirements of pathogens has the possibility of identifying new, specific, therapeutic targets. In *C. neoformans*, biosynthesis is one aspect of amino acid acquisition that has been studied and linked to pathogenesis in the last 10 years. Several pathways have been associated with virulence attenuation in animal models of infection, which would serve as putative molecular targets for antifungal development [126]. Commercial inhibitors of anthranilate synthase component II and tryptophan synthase were tested and are active against both *C. neoformans* and *C. gattii*, demonstrating the potential application of inhibitors directed at the tryptophan biosynthetic pathway. The data suggest that the *C. neoformans* tryptophan biosynthetic pathway is an excellent pharmacological target. Furthermore, inhibitors of this pathway cause *Cryptococcus* growth arrest in vitro [124]. 

The isoleucine and valine biosynthetic enzyme acetolactate synthase (*Ilv2*p) is also an attractive antifungal drug target, since the isoleucine and valine biosynthetic pathway is not present in mammals, *S. cerevisiae ilv2*Δ mutants do not survive in vivo, *C. neoformans ilv2* mutants are avirulent, and both *S. cerevisiae* and *C. neoformans ilv2* mutants die upon isoleucine and valine starvation [183] The conserved subset of amino acid biosynthetic enzymes in fungi, not present in humans, offer exciting potential as an unexploited class of antifungal drug targets. Threonine biosynthesis has been shown to be essential in *Cryptococcus neoformans* and in other yeasts and fungi. Through examination of the survival of *Saccharomyces cerevisiae* homoserine kinase (thr1) and threonine synthase (thr4) mutants in mice demonstrated that threonine biosynthetic enzymes could be targeted for new antifungal drug candidates [184]. In addition, as described above, many cryptococcal lipids and their biosynthesis are distinct to mammalian cells and may be targeted for therapy [185].

Thus, as a soil and botanic dwelling basidiomycete yeast, *Cryptococcus* has evolved a broad range of nutrient acquisition strategies. Many, if not most, of these strategies also appear to be beneficial to infection of the host. While thermotolerance is the primary feature that sets the pathogenic cryptococci apart [186], related species of cryptococci are an important resource in the understanding of the *C. neoformans* and *C. gattii*. Considering nutrition acquisition is also a useful exercise when studying experimental models of infection, not only because nutrient retention is a proven strategy of immune systems, but because human pathogenic cryptococci did not evolve to be animal pathogens. Finally, there are many experimental animal models that represent the environmental predator–prey relationships of the cryptococcal ecological niche. While a number of these have been used to a limited extent [108,173,179,181,187,188,189], there is significant scope for using such models in understanding this fatal fungal pathogen.
